# Mandibular Fracture Associated with a Dentigerous Cyst: Report of a Case and Literature Review

**DOI:** 10.15171/joddd.2015.035

**Published:** 2015-09-16

**Authors:** Maryam Kouhsoltani, Ali Hossein Mesgarzadeh, Monir Moradzadeh Khiavi

**Affiliations:** ^1^Dental and Periodontal Research Center, Tabriz University of Medical Sciences, Tabriz, Iran; ^2^Assistant Professor, Department of Oral and Maxillofacial Pathology, Faculty of Dentistry, Tabriz University of Medical Sciences, Tabriz, Iran; ^3^Associate Professor, Department of Oral and Maxillofacial Surgery, Faculty of Dentistry, Tabriz University of Medical Sciences, Tabriz, Iran; ^4^Associate Professor, Department of Oral and Maxillofacial Pathology, Faculty of Dentistry, Tehran University of Medical Sciences, Tehran, Iran

**Keywords:** Dentigerous cyst, odontogenic cyst, pathologic fracture

## Abstract

***Background and aims***. Pathological fractures are rare in the maxillofacial region and account for less than 2% of all fractures in this site. They are defined as fractures that take place when bone has been weakened by an underlying pathologic process. Among all pathoses, cysts (although so common in the maxillofacial region) constitute a very small part. Here we report a case of a dentigerous cyst in a 38-year-old man.The cyst was associated with a mandibular second premolar tooth and resulted in a pathologic fracture. Excision of the lesion was performed and bony union was observed after 6 months. In the literature review, only one case of dentigerous cyst causing pathologic fracture was found. In addition to the report of the present case, pathologic fractures associated with all types of odontogenic cysts (totally just 12 cases) are reviewed in this article to provide a comprehensive and detailed collection.

## Introduction

Pathologic fracture is usually defined as a type of fracture which is caused by an underlying pathologic lesion. The pathologic process weakens the bone and the fracture results from normal function or inadequate trauma. Pathologic bone fractures are uncommon in general and rarely occur within facial bones. Pathologic fractures of the maxillofacial region most frequently result from osteoradionecrosis and it is very interesting that cysts, which are so common in this area, rarely cause fractures.^1-4^

Dentigerous cysts are odontogenic cysts that are associated with the crowns of unerupted teeth. The pathogenesis of these cysts is unknown and they are believed to develop by accumulation of fluid between reduced enamel epithelium and tooth crown. Dentigerous cysts most frequently involve mandibular third molars and are generally seen in patients between 10 and 30 years of age.^5-6^ These cysts are frequently asymptomatic and may exist for several years without being discovered. In clinical examination, missing teeth and probably areas of hard swellings are observed, but there is usually no associated pain or discomfort.^7^A dentigerous cyst may grow to a significant size and result in cortical expansion of the bone and facial asymmetry, but very rarely predisposes the patient to a pathologic fracture.^5-7^Typically, a dentigerous cyst shows a unilocular radiolucency with well-defined and sclerotic borders that is associated with the crown of an unerupted tooth.^8^

We report a case of dentigerous cyst in a 38-year-old man. The cyst was associated with an unerupted mandibular second premolar tooth and resulted in pathologic bone fracture. Excision of the cyst was performed and after 6 months bony union was radiographically observed. Moreover, the literature reports of the pathologic fractures associated with all types of odontogenic cysts (totally 12 cases, including only one case of dentigerous cyst) are reviewed in the present article.

## Case Report

A 38-year-old man presented with an asymptomatic mandibular mass. He had been slightly punched in his face 2 days before visiting the Department of Oral Surgery, Faculty of Dentistry, Tabriz University of Medical Sciences. His chief complaint was pain and swelling in the jaw. The patient had no complaints before, except some degree of paresthesia in the region. The medical history of the patient revealed no systemic, endocrine or metabolic disorders. On extraoral examination, no abnormality was detected except slight asymmetry of the face. On palpation, crepitation was found in the inferior border of the left mandible. Intraoral examination revealed missing lower left second premolar, and hard swelling and some degree of erythema in the left posterior mandible area. The patient declared that the lower left first molar had been extracted about five years previously. However, some degree of erythema was observed on the edentulous mucosa between the lower left first premolar and lower left second molar.

**Figure 1.**
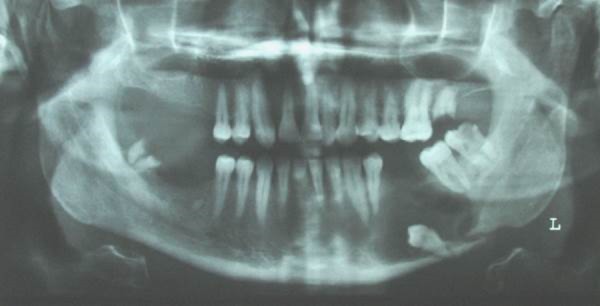
Panoramic radiograph showing a cystic lesion around an impacted second premolar. A fracture is also evident in the inferior border of the mandible.

Radiographically, a well-defined radiolucency was identified in the body of the mandible on the left side. The radiolucency extended from the first premolar region to the second molar area. It seemed to have destroyed bone almost from the edentulous alveolar crest to the inferior border of the mandible. A small ill-defined area was observed in the anterior border of the lesion. The lesion was associated with an obvious bony fracture in the inferior border of the mandible. The relatively large size of the lesion had pushed the lower left second premolar to the inferior border of the mandible (Figure 1).

**Figure 2.**
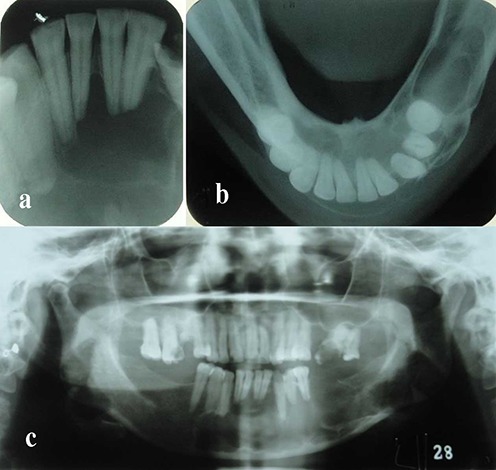
Histopathologic features (a-c): dentigerous cyst lined by flat nonkeratinizing epithelium (H&E, ×400).

**Figure 3.**
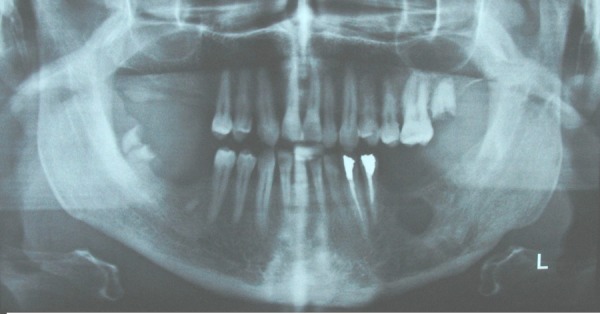
Bony union at the fracture site is seen on the follow-up panoramic radiograph.

The differential diagnoses of odontogenic keratocyst, dentigerous cyst, ameloblastoma and calcifying epithelial odontogenic tumor were considered for the relatively large and well-defined pericoronal radiolucency. Unbelievably, the histopathologic evaluation of the incisional biopsy material led to the diagnosis of ossifying fibroma. The patient subsequently underwent surgery for complete removal of the lesion (excisional biopsy) under local anesthesia and via an intraoral approach. Simultaneously, interdental fixation was carried out. Histopathologic examination of multiple sections revealed a cystic lesion lined by flattened nonkeratinized epithelium. Typical two to four layers of the epithelium of dentigerous cyst and flat interface of epithelium and connective tissue were observed in most parts (Figure 2). Infiltration of chronic inflammatory cells was observed in the underlying connective tissue. Epithelial hyperplasia caused by inflammation was observed in some parts. Based on these findings, the diagnosis of dentigerous cyst was made. Two weeks after surgery, the patient asked to remove the arch bars and further treatment and fixation of the fracture site were not applicable. Fortunately, bony union was radiographically observed after 6 months (Figure 3), and the patient remained symptom-free over a postoperative period of 2 years.

## Discussion

In the craniofacial region, the most common causes of fractures are traumas, often from motor vehicle accidents or interpersonal violence. Presence of pathoses in the bone can considerably reduce the tensile strength and encourage dissemination of the fracture (from inadequate trauma) along the least-resistant path. This type of fracture is usually referred to as "pathologic fracture". In the case presented here, the patient had been slightly punched in his face 2 days before visiting our department. However, this minor trauma had resulted in a pathologic bone fracture. As an explanation, the pathologic process had weakened the bone, resulting in fracture with the minimal trauma. Pathologic fractures of the jaws may result from severe atrophy of edentulous alveolar ridges, osteoradionecrosis, osteomyelitis, bisphosphonate-related osteonecrosis, benign and malignant tumors, metastatic neoplasms or cysts.^1-4,9^ Pathologic fractures associated with cysts have been very rarely reported and, as far as we could determine, only 12 satisfactory cases of pathologic fractures associated with odontogenic cysts have been reported in the literatures (Table 1).^1-3,10-13^, We could find only 2 cases of follicular (dentigerous) cysts resulting in pathologic fracture, but one of them was ruled out because it was not associated with an unerupted tooth. However, dentigerous cyst is the most common type of developmental odontogenic cysts. This cyst is the second most common odontogenic cyst after radicular or periapical cyst.

**Table 1:**
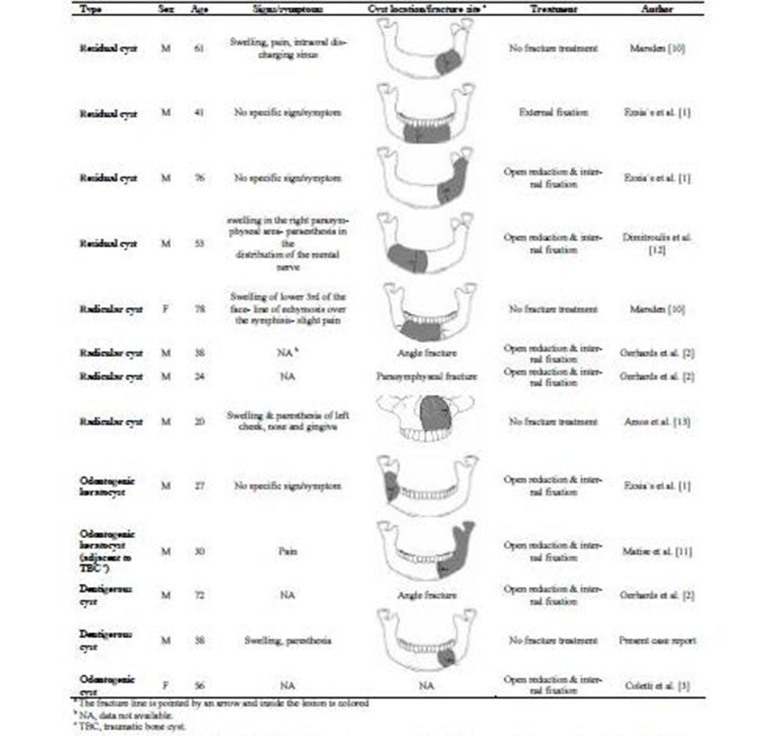
Reports of odontogenic cysts associated with pathologic fractures

Radicular cyst develops at the apex of a nonvital tooth and is the most commonly diagnosed odontogenic cyst, followed respectively by dentigerous cyst, odontogenic keratocyst and residual cyst in most studies.^14-16^Among the 13 case reports (including the case presented in this article), radicular and residual cysts were the most common types of odontogenic cysts that were associated with pathologic fractures (each about 33%). Pathologic fractures related to the residual cysts were abnormally high in comparison with their prevalence, which can be explained by the trauma during tooth extraction or the atrophy of the alveolar ridge due to tooth loss. Interestingly, odontogenic keratocysts, despite the infiltration in the medullary cavity and relatively large sizes, are not associated with high rate of pathologic fractures. Dentigerous cysts accounted for approximately 16.6% of odontogenic cysts associated with pathologic fractures.

Mean age was 47.2 years, ranging between 20 and 78 years. Typically, odontogenic cysts have a slight male predilection.^5-6^ However, almost all patients were male in 13 case reports summarized in Table 1(M/F: 11/2). This significant male predominance may be explained by the fact that men, in general, are more susceptible to trauma and are less careful about their health, which may lead to large cysts and consequently weak bones vulnerable to fractures.

There is no agreement in the literature about the site predilection of odontogenic cysts. Previous studies have reported both the maxilla and the mandible as the most frequently affected sites by odontogenic cysts. In most studies, the most commonly affected site was the anterior maxilla.^16-19^ However, pathologic fractures of the jaws almost always involve the mandible. Among the odontogenic cysts that were associated with pathologic fractures, 12 cases occurred in the mandible and only 1 case occurred in the maxilla.

The anatomical distribution of mandibular fractures cannot be explained only on the basis of mandibular anatomy. It is largely dependent on various clinical factors, such as type, magnitude, or direction of impacting force, presence of soft tissue bulk, three-dimensional biomechanical properties of the bone like bone density, and normal or pathologic anatomic structures creating lines of relative weakness within the bone. Generally, about 25–33% of mandibular fractures are angle fractures.^20^ Among the pathologic fractures included in the present study, the most common fracture sites were parasymphysis and body of the mandible (each site involved in about 33% of cases), followed respectively by angle the of the mandible (25%) and lateral wall of the maxillary sinus (about 8%). A case of bilateral fracture associated with radicular cyst has been reported in a 78-year-old woman.

Typically, the patients with odontogenic cysts are asymptomatic and the lesions are discovered on radiographic examinations or when films are taken to find out the reason for failure of a tooth to erupt. Odontogenic cysts can reach a considerable size, and large cysts may be associated with swelling of the bone and mild sensitivity in the area involved.^5-6^The most common sign/symptom associated with pathologic bone fracture as a result of odontogenic cyst was swelling of the affected bone. This can be explained by the fact that most cases were large cysts which resulted in expansion and fracture of the involved bone. Pain and paresthesia has been reported less commonly.

Although dentigerous cysts can involve any unerupted tooth, they are most frequently associated with mandibular third molars. In the case presented in this article, a mandibular second premolar was involved. All cases of odontogenic keratocyst were located in the posterior mandible and associated with impacted third molars.

In most cases open reduction and internal fixation were carried out after cyst enucleation or marsupialization. When a significant amount of bone was lost, resection of the involved bone was performed to reconstruct and fix the area. In the present case, interdental fixation was performed after enucleation of the cyst due to patient preference and financial state. However, the preferred treatment was reconstruction and fixation of the area due to significant bone loss.

## Conclusion

Based on the results of this study, pathologic fractures that are associated with odontogenic cysts are rare. They almost always involve the mandible and the incidence is considerably higher in men. Among the cases included in the present study, the most common fracture sites were parasymphysis and body of the mandible and the most common sign/symptom was swelling of the affected bone. Radicular and residual cysts were the most common types of odontogenic cysts associated with pathologic fractures. As a conclusion, regular follow-up visits with radiographic images are required in cases of symptom-free impacted teeth and periapical pathoses to avoid untoward effects.
